# Diagnostic performance and clinical impact of ^18^F-AlF-NOTA-octreotide in a large cohort of patients with neuroendocrine neoplasms: A prospective single-center study

**DOI:** 10.7150/thno.96762

**Published:** 2024-05-19

**Authors:** Guozhu Hou, Xin Cheng, Yi Yang, Dongbing Zhao, Guiqi Wang, Hong Zhao, Rong Zheng, Xuejuan Wang, Xiaoyuan Chen, Yihebali Chi, Jingjing Zhang

**Affiliations:** 1 Department of Nuclear Medicine (PET-CT Center), National Cancer Center/National Clinical Research Center for Cancer/Cancer Hospital, Chinese Academy of Medical Sciences and Peking Union Medical College, Beijing, China.; 2 Department of Hepatobiliary Surgery, National Cancer Center/National Clinical Research Center for Cancer/Cancer Hospital, Chinese Academy of Medical Sciences and Peking Union Medical College, Beijing, China.; 3 Department of Pancreatic and Gastric Surgery, National Cancer Center/National Clinical Research Center for Cancer/Cancer Hospital, Chinese Academy of Medical Sciences and Peking Union Medical College, Beijing, China.; 4 Department of Endoscopy, National Cancer Center/National Clinical Research Center for Cancer/Cancer Hospital, Chinese Academy of Medical Sciences and Peking Union Medical College, Beijing, China.; 5 Department of Diagnostic Radiology, Yong Loo Lin School of Medicine and Faculty of Engineering, National University of Singapore, Singapore.; 6 Clinical Imaging Research Centre, Centre for Translational Medicine, Yong Loo Lin School of Medicine, National University of Singapore, Singapore.; 7 Theranostics Center of Excellence (TCE), Yong Loo Lin School of Medicine, National University of Singapore, 11 Biopolis Way, Helios, Singapore 138667; 8 Nanomedicine Translational Research Program, NUS Center for Nanomedicine, Yong Loo Lin School of Medicine, National University of Singapore, Singapore.; 9 Departments of Surgery, Chemical and Biomolecular Engineering, and Biomedical Engineering, Yong Loo Lin School of Medicine and College of Design and Engineering, National University of Singapore, Singapore; 10 Institute of Molecular and Cell Biology, Agency for Science, Technology, and Research (A*STAR, National University of Singapore, 61 Biopolis Drive, Proteos, Singapore, Singapore; 11 Department of Medical Oncology, National Cancer Center/National Clinical Research Center for Cancer/Cancer Hospital, Chinese Academy of Medical Sciences and Peking Union Medical College, Beijing, China.

**Keywords:** neuroendocrine neoplasms (NENs), ^18^F-AlF-OC, PET/CT, somatostatin receptors (SSTRs), clinical practice, contrast-enhanced CT/MRI

## Abstract

**Purpose:** Somatostatin receptor imaging with ^18^F-AlF-NOTA-octreotide (^18^F-AlF-OC) has shown promising performance in neuroendocrine neoplasms (NENs). In this study, we aim to investigate the diagnostic performance and clinical impact of ^18^F-AlF-OC in a large prospective cohort of patients with NEN.

**Methods:** Between January 2023 and November 2023, a total of 219 patients with confirmed or suspected NEN were enrolled prospectively and underwent ^18^F-AlF-OC PET/CT at 2 h post-injection. The primary endpoint was the diagnostic performance, including sensitivity, specificity, and accuracy. An additional primary endpoint was the impact of ^18^F-AlF-OC on clinical management. The reference standard was based on the results of histopathology or radiological follow-up.

**Results:** 205 patients were included in the final analysis. The patient-level sensitivity, specificity, and accuracy of ^18^F-AlF-OC PET/CT compared with contrast-enhanced CT/MRI were 90.5% vs. 81.8%, 93.1% vs. 71.1%, and 91.2% vs. 79.4%, respectively. 26 patients had tiny gastrointestinal NENs (smaller than 1 cm in diameter). The patient-based sensitivity of ^18^F-AlF-OC PET/CT and contrast-enhanced CT/MRI were 61.5% (16/26) and 37.5% (9/24), respectively. The smallest diameter of gastrointestinal NEN detected by ^18^F-AlF-OC PET/CT was 0.6 cm in the rectum, 0.3 cm in the stomach, and 0.5 cm in the duodenum. ^18^F-AlF-OC PET/CT results led to changes in clinical management in 19.5% of patients (40/205), owing mainly to new or unexpected findings compared to contrast-enhanced CT/MRI.

**Conclusion:** ^18^F-AlF-OC PET/CT demonstrated great diagnostic performance in patients with NEN, particularly for detecting tiny gastrointestinal NEN. Furthermore, ^18^F-AlF-OC PET/CT impacted the therapeutic management in 19.5% of patients. Our results further validate the role of ^18^F-AlF-OC as a somatostatin receptor imaging tracer in clinical practice.

## Introduction

Neuroendocrine neoplasms (NENs) represent a heterogeneous group of tumors originating from cells of the diffuse neuroendocrine system. Well-differentiated NENs are characterized by the overexpression of somatostatin receptors (SSTRs) on the cell surface, which thereby provides an ideal target for molecular imaging and therapy using radiolabeled somatostatin analogs (SSAs). Currently, Gallium-68 labeled SSA PETCT, including ^68^Ga-DOTATATE, and ^68^Ga-DOTATOC are standard functional imaging modalities in the management of NENs 1. Nevertheless, the application of ^68^Ga-SSAs in clinical practice is limited by the high production cost, limited availability, and low activity yield pre-elution of ^68^Ge/^68^Ga-generators and the short half-life (*t*_1/2_, 67.8 min) of ^68^Ga. In this context, Fluorine-18 labeled SSAs are emerging as a promising alternative for SSTR imaging. Fluorine-18, the most widely used PET radionuclide, has a high production yield and favorable half-life (*t*_1/2_, 109.8 min). Furthermore, the lower positron energy of ^18^F compared to ^68^Ga enables a shorter positron linear range in tissue (2.3 mm), which could potentially improve spatial resolution and better image quality 2. ^64^Cu-DOTATATE is commercially available in the USA. A previous study reported that ^64^Cu-DOTATATE has advantages over ^68^Ga-DOTANOC in the detection of lesions in NET patients 3. ^64^Cu has a relatively long half-life of 12.7 h, which has some advantages in distribution, but it leads to increased radiation exposure.

Recently, a fluorine-18-labeled SSA, namely ^18^F-Al-1,4,7-triazacyclononane-1,4,7-tri-acetate-octreotide (^18^F-AlF-NOTA-octreotide; ^18^F-AlF-OC), has shown promising performance for imaging NEN.^ 18^F-AlF-OC can be rapidly and robustly synthesized using a chelator-based Al^18^F-method 4, 5. Earlier clinical translations of ^18^F-AlF-OC in healthy volunteers and NEN patients have reported favorable biodistribution, dosimetry profile, and lesion targeting 6, 7. Previously reported comparisons of ^18^F-AlF-OC with ^68^Ga-DOTATATE in small groups of patients with NEN (n = 6, 10, 20, respectively) found that ^18^F-AlF-OC have similar lesion detectability and tumor uptake 7-9. A recent prospective study by Pauwels *et al.* reported that ^18^F-AlF-OC is non-inferior and even superior to ^68^Ga-DOTATATE/NOC, in a cohort of 75 NEN patients 10. Recently, Chen *et al.* compared ^18^F-AlF-OC and contrast-enhanced CT/MRI in a retrospective cohort of 93 patients with confirmed or suspected NEN. Their results showed that ^18^F-AlF-OC has a superior diagnostic performance over contrast-enhanced CT/MRI by demonstrating higher sensitivity and accuracy, supporting its feasibility as an alternative SSTR-targeted PET/CT tracer 11. These results are encouraging. Still, there is a lack of prospective studies investigating the diagnostic performance of ^18^F-AlF-OC in a large cohort of patients with NEN, and to our knowledge, there are no published data on the impact of ^18^F-AlF-OC PET/CT on clinical management. The purpose of this prospective study was to explore the diagnostic performance and clinical impact of ^18^F-AlF-OC in a large cohort of patients with NENs.

## Materials and Methods

### Study Population

This study was approved by the Institutional Review Board of our hospital and performed in accordance with the Declaration of Helsinki and is registered at ClinicalTrials.gov (registration number: NCT05749289). All subjects signed a written informed consent. A total of 219 patients were prospectively enrolled in this study between January 2023 and November 2023. The inclusion criteria were as follows: 1) age ≥ 18 years; 2) patients with pathologically confirmed NEN (biopsy or surgery) in metastasis, recurrence, or primary tumor; 3) patients with clinically suspected NEN (elevated levels of tumor markers, clinical presentation or image-based suspicion) enrolled for diagnostic evaluation. The exclusion criteria were: 1) pregnant or lactating women; 2) the presence of other malignant tumors; 3) inability to complete the study.

### ^18^F-AlF-OC Preparation and PET/CT Acquisition

^18^F-AlF-OC was synthesized as previously described 6, 7, 12, under good manufacturing practice guidelines. The yield of the synthesis was approximately 20%. The radiochemical purity of the final product was over 95%. All patients were intravenously injected with ^18^F-AlF-OC at a dose of 257.5∼444 MBq (average, 336.4 ± 48.0 MBq). PET/CT imaging (from mid-thigh to vertex) was performed at 2 h after injection, which was previously identified as the optimal time point for imaging for this tracer 7. An unenhanced low-dose CT scan was obtained for attenuation correction and anatomical reference, followed by a PET scan at 2 min/bed position. All scans were performed on dedicated PET/CT scanners (Discovery 690; GE Healthcare). PET images are acquired from the head to the upper femur (usually 7-8 bed position) in three-dimensional mode at 2 min/frame. The VPFX-S algorithm (2 iterations, 24 subsets, 4 mm Gaussian post filter) was used for image reconstruction. Spiral CT was obtained with a tube voltage of 120 kV, tube current of 150 mA, 3.75 mm of slice thickness, 1.375 of pitch, and 0.8 s of rotation speed.

### Image Analyses

PET/CT images were analyzed by 2 experienced nuclear medicine physicians on Advantage Workstation (version 4.6; GE Healthcare). Any focal ^18^F-AlF-OC uptake that was higher than the background activity and could not be explained by physiologic uptake was considered a positive lesion. A PET scan was considered positive if at least one positive lesion was detected, otherwise the scan was considered negative. Tumor uptake was semi-quantified by the maximal standardized uptake value (SUVmax). All CT and MRI images were registered in the Picture Archiving and Communication Systems (PACS) software and were analyzed by an experienced radiology physician. The reference standard was based on the histopathological results (n = 143) or radiographic follow-up (n = 62; range, 4.5∼14 months, median, 8.5 months).

### Evaluation of Impact of ^18^F-AlF-OC PET/CT on Clinical Management

Changes in clinical management related to findings on ^18^F-AlF-OC PET/CT were recorded after retrospective review of patient charts. This was done by documenting clinical management based on contrast-enhanced CT/MRI and based on ^18^F-AlF-OC PET/CT.

### Statistical Analyses

Statistical analyses were performed using SPSS Statistics for Windows version 21.0 (IBM Corp, Armonk, NY, USA). Descriptive statistics were used. The sensitivity, specificity, and accuracy of ^18^F-AlF-OC PET/CT and contrast-enhanced CT/MRI were calculated and compared on a per-patient basis. For tiny gastrointestinal NENs, the sensitivity of ^18^F-AlF-OC PET/CT and contrast-enhanced CT/MRI were compared on a per-patient and per-lesion basis. Categorical variables are presented as numbers and percentages.

### Data availability

The data generated in this study are available within the article. Further data generated in this study are not publicly available due to patient privacy but are available upon reasonable request from the corresponding author.

## Results

### Patient Characteristics

Between January 2023 and November 2023, 219 patients with confirmed or suspected NENs were enrolled to undergo ^18^F-AlF-OC PET/CT. The diagnosis could not be confirmed in 14 patients. Thus, 205 patients (111 men and 94 women; age range, 20 to 94 y, median, 55 y) were included in the final analysis. The patients' demographics and characteristics are summarized in Table 1. Of the 205 patients, 192 suffered from pathologically confirmed NENs and underwent ^18^F-AlF-OC PET/CT for detecting metastasis, recurrence, or the primary tumor; 13 were patients with suspected NENs who underwent ^18^F-AlF-OC PET/CT for diagnostic evaluation. For the 13 patients with suspected NENs, histopathological results confirmed NENs in 9 cases. The remaining 4 cases were diagnosed with other conditions: pancreatic cancer (n = 1), gastric adenoma (n = 1), jejunal stromal tumor (n = 1), and follicular dendritic cell tumor (n = 1). Of the total 201 patients with pathologically confirmed NENs, 74 were grade 1 (G1), 90 were grade 2 (G2), 21 were grade 3 (G3), and 6 were neuroendocrine carcinomas (NEC) according to the 2019 World Health Organization (WHO) Classification of Digestive System Tumors, 9 were paragangliomas, and the grading information was not available for 1 patient. Of the 205 patients, 70 were imaged at diagnosis for diagnostic evaluation, or detecting possible metastasis or primary tumor, and 135 patients were imaged for restaging. The primary tumor was most frequently found in the pancreas (46/201, 22.9%), followed by the stomach (39/201, 19.4%). Other primary sites included the rectum (31/201, 15.4%), small intestine (25/201, 12.4%), thymus (17/201, 8.5%), lung (15/201, 7.5%), and others (10/201, 5.0%). 9 patients (9/201, 4.5%) suffered from paragangliomas. In the remaining 9 patients (9/201, 4.5%), the primary site was unidentified.

### Diagnostic Performance

Of the 205 patients, 137 were positive, and 68 were negative on ^18^F-AlF-OC PET/CT. According to the reference standard, there were 133 true positive and 54 true negative cases, 4 false positive and 14 false negative cases. With regard to the false-positive cases, 2 patients had focal ^18^F-AlF-OC uptake because of inflammatory changes, and 2 patients had focal uptake in non-NEN lesions (one pancreatic cancer tumor and one follicular dendritic cell tumor). As for the false-negative cases, 10 NEN lesions with less than 1 cm in diameter were missed. For the rest, the NEN was of high grade (NEC, Ki-67 90%) in 1 case and of low grade (G1) in 3 cases. The patient-level sensitivity, specificity, and accuracy of ^18^F-AlF-OC PET/CT were 90.5% (133/147), 93.1% (54/58), and 91.2% (187/205), respectively, in comparison to contrast-enhanced CT/MRI with sensitivity of 81.8% (112/137), specificity of 71.1% (27/38), and accuracy of 79.4% (139/175), respectively (Table 2). The diagnostic performance of ^18^F-AlF-OC PET/CT according to the WHO grade is shown in Table 3. For G2 patients, who made up the largest proportion, ^18^F-AlF-OC PET/CT exhibited a sensitivity of sensitivity of 100% (68/68), specificity of 90.9% (20/22), and accuracy of 97.8% (88/90), respectively.

**Figure 1:**
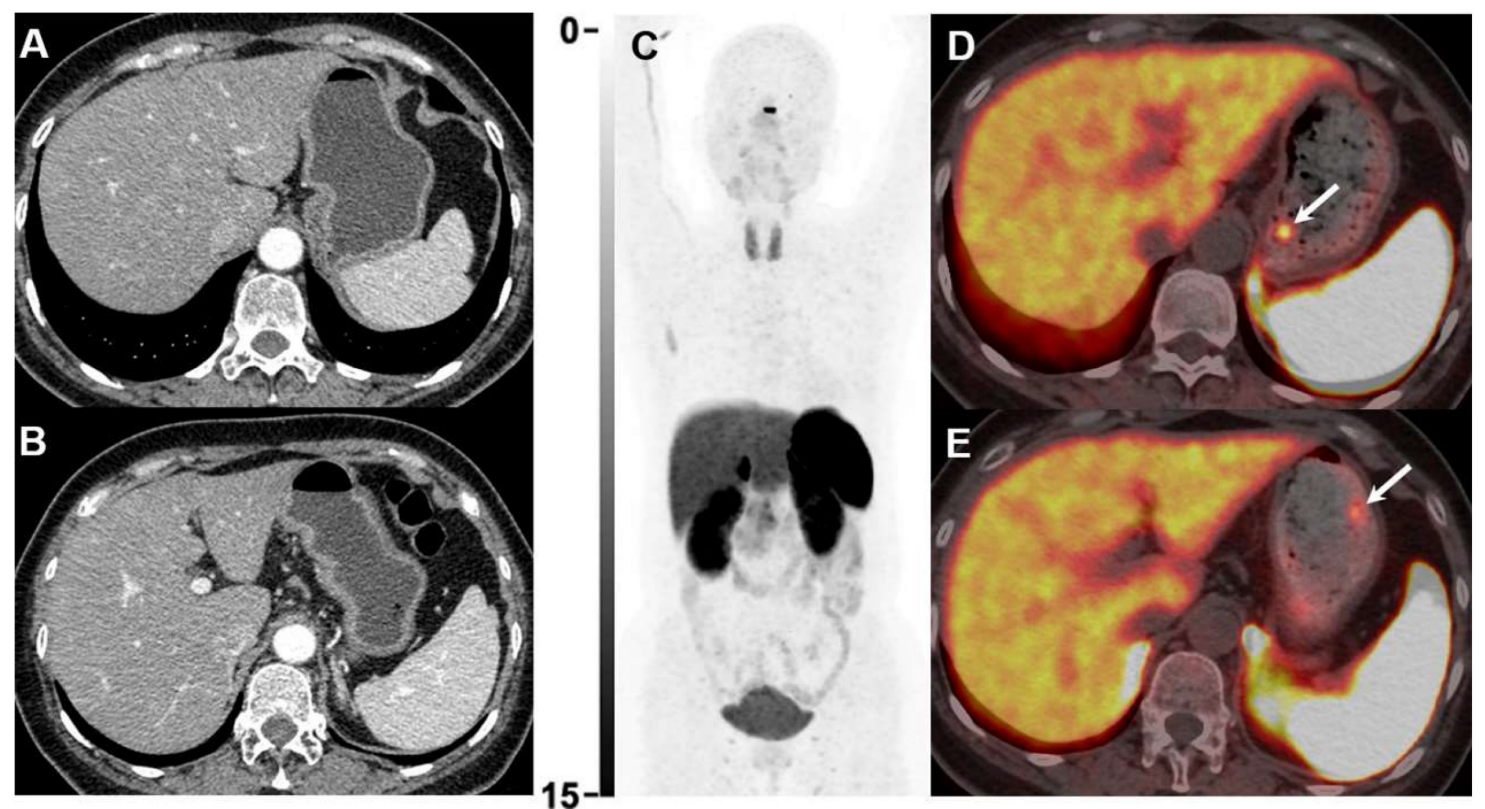
Gastroscopy revealed 2 tiny gastric lesions (both 0.3 cm in diameter) in a 49-year-old female patient, highly suspicious of NEN. Contrast-enhanced CT was negative (A and B). ^18^F-AlF-OC PET/CT (C-E) was then performed for diagnostic evaluation and detecting possible metastases. ^18^F-AlF-OC PET/CT successfully detected the 2 gastric lesions with increased uptake (**D**, SUVmax=6.9, **E**, SUVmax=3.9, white arrows). The patient was subsequently treated with endoscopic resection, and histopathology confirmed the diagnosis of NEN (G1, Ki-67 1%).

In 26 patients, tiny gastrointestinal NENs were identified at endoscopy. ^18^F-AlF-OC PET/CT was thereafter recommended for detecting possible metastasis (pre-treatment staging). The gastrointestinal NENs were all diagnosed by histopathology (gastric NEN in 14, duodenal NEN in 6, rectal NEN in 6). There were in total 41 gastrointestinal NEN tumors in these 26 patients, the diameter of which ranged from 0.1 to 0.9 cm (median, 0.6 cm). On a per-patient level, the sensitivity of tiny NEN lesions by ^18^F-AlF-OC PET/CT and contrast-enhanced CT/MRI was 61.5% (16/26) and 37.5% (9/24), respectively (Figure 1). On a per-lesion level, the sensitivity of ^18^F-AlF-OC PET/CT and contrast-enhanced CT/MRI was 53.7% (22/41) and 35.1% (13/37), respectively. The smallest diameter of gastrointestinal NEN detected by ^18^F-AlF-OC PET/CT was 0.6 cm in the rectum, 0.3 cm in the stomach, and 0.5 cm in the duodenum. In addition, ^18^F-AlF-OC PET/CT detected lymph node metastases in 6 patients.

### Impact of ^18^F-AlF-OC PET/CT on Clinical Management

^18^F-AlF-OC PET/CT results led to changes in therapeutic management in 40 patients (19.5%, 40/205), owing mainly to additional or unexpected findings compared to contrast-enhanced CT/MRI. In most instances, the new treatment that patients received after ^18^F-AlF-OC PET/CT was SSAs (n = 11). In 2 patients, SSA treatment followed by peptide receptor radionuclide therapy (PRRT) was recommended after ^18^F-AlF-OC PET/CT. One patient received SSAs and targeted therapy after ^18^F-AlF-OC PET/CT. Targeted therapy was initiated in 8 patients, and chemotherapy in 5 patients. Immunotherapy combined with chemotherapy was initiated in 1 patient. In 2 patients, ^18^F-AlF-OC PET/CT showed progression of the disease after the end of chemotherapy, prompting another chemotherapy regimen to be provided thereafter.

In 7 patients, surgical treatment was recommended after ^18^F-AlF-OC PET/CT. In 4 patients, the recommendation was based on the identification of the previously unknown site of primary NEN or confirmation of the suspected site. In the remaining 3 patients, surgical resection of metastatic lesions was recommended as^ 18^F-AlF-OC PET/CT identified limited and surgically resectable disease. In 2 patients, ^18^F-AlF-OC PET/CT confirmed the suspected liver metastases, which was unequivocal on MRI, and showed that the disease was limited to the liver. In the other patient, ^18^F-AlF-OC PET/CT detected solitary lymph node metastasis in the pelvis, which was missed by contrast-enhanced CT.

**Figure 2:**
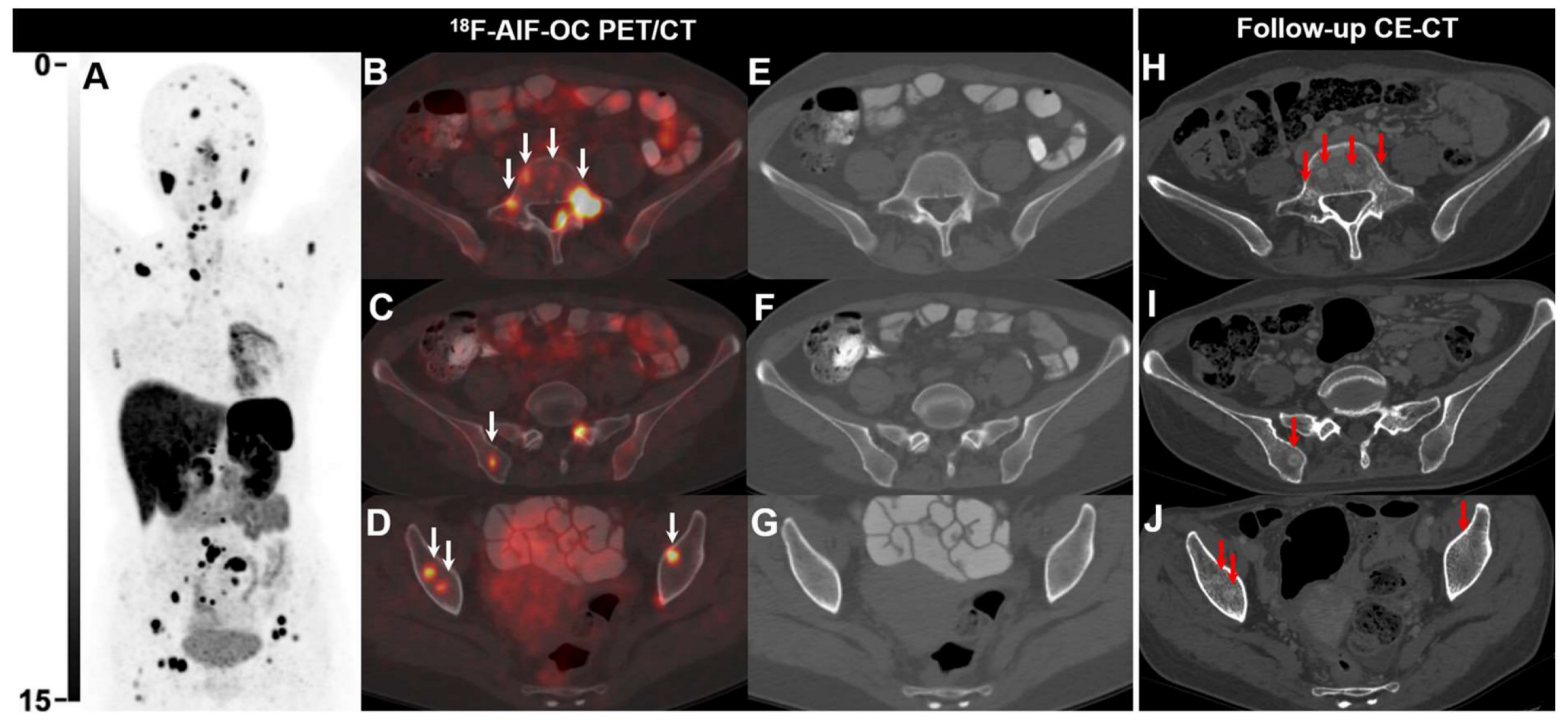
^ 18^F-AlF-OC PET/CT images of a 39-y-old female patient with lung NEN (G2, Ki-67 8%). The patient was previously treated with chemotherapy and radiotherapy. ^18^F-AlF-OC PET/CT was performed for posttreatment restaging and revealed multiple unexpected bone lesions, suggesting progressive disease (**A-G**). Note that the bone lesions detected on ^18^F-AlF-OC PET/CT were not visible on CT as there were no morphological and density abnormalities (**E-G**). Subsequently, the patient received somatostatin analogs and targeted therapy (Sulfatinib). 8 months later, follow-up CE-CT revealed multiple osteoblastic changes (red arrows,** H-J**), corresponding to the bone lesions detected on ^18^F-AlF-OC PET/CT.

In 11 patients, treatment with an antiresorptive agent (bisphosphonate or denosumab) was initiated due to newly diagnosed bone metastases by ^18^F-AlF-OC PET/CT. In 8 patients, the precise management change was unclear from the records. An example of ^18^F-AlF-OC PET/CT findings leading to a change in management is shown in Figure 2.

## Discussion

In this prospective study, we assessed the diagnostic performance of ^18^F-AlF-OC and its impact on therapeutic management in a large cohort of NEN patients. In the present study, ^18^F-AlF-OC PET/CT demonstrated higher sensitivity (90.5% *vs.* 81.8%) and diagnostic accuracy (91.2% *vs.* 79.4%) for NENs than contrast-enhanced CT/MRI. Our large-cohort-based results support those of Chen *et al.*, who retrospectively evaluated the diagnostic value of ^18^F-AlF-OC PET/CT in 93 NEN patients 11. Using histopathology or imaging follow-up as the reference standard, they also found that ^18^F-AlF-OC PET/CT has better sensitivity and accuracy than contrast-enhanced CT/MRI. The diagnostic superiority of ^18^F-AlF-OC PET/CT over contrast-enhanced CT/MRI is of great clinical significance, including its impact on clinical management, which will be discussed later. We noticed that the patient-based sensitivity observed in the present study was slightly lower than theirs (90.5% *vs.* 96.3%). This difference might probably be caused by the big difference in the sample size between studies. Although their study included a total of 93 patients with suspected or confirmed NENs, the per-patient diagnostic performance was analyzed only in 45 patients with suspected NENs, a much smaller number than the 205 patients analyzed for per-patient diagnostic performance in the present study. It is also worth noting that 26 patients in our cohort had tiny NEN lesions (smaller than 1cm in diameter), and 10 of them had false negative results on ^18^F-AlF-OC PET/CT, which also made up the majority of the false negative cases (10/14) in this study. We suspected that the unexpected inclusion of patients with tiny NENs could explain the slightly lower sensitivity observed in our study.

^68^Ga-DOTATATE PET/CT is the current standard of molecular imaging for NEN patients. The diagnostic performance of ^18^F-AlF-OC PET/CT observed in our study is comparable to that of ^68^Ga-DOTATATE PET/CT in the available literature reports. A meta-analysis from 2016 encompassed 10 studies with 465 patients 13. The per-patient sensitivity of ^68^Ga-DOTATATE ranged from 81% to 100%, and specificity 86% to 100% 14-23. The pooled estimates of sensitivity and specificity of ^68^Ga-DOTATATE were 90.9% and 90.6%, respectively. Several previous studies comparing ^18^F-AlF-OC PET/CT with ^68^Ga-DOTATATE PET/CT have revealed similar lesion detectability and tumor uptake 7-10. It is therefore not surprising that on a patient basis, our study also did not show any notable difference in the diagnostic performance between ^18^F-AlF-OC PET/CT and previously reported results of ^68^Ga-DOTATATE PET/CT.

The majority of gastrointestinal NENs are less than 1 cm in size at the time of diagnosis 24-28; therefore, it is important to provide an imaging modality able to detect such lesions, which pose a challenge for diagnostic imaging. There are reports of tiny NEN cases detected by SSTR PET/CT in the literature; however, the sensitivity of SSTR PET/CT in this scenario specifically has not been investigated before. Our results revealed that the patient-based sensitivity of ^18^F-AlF-OC PET/CT for detecting tiny gastrointestinal NEN was 61.5%. While this sensitivity is lower than its sensitivity for larger NENs, it can still be considered quite favorable and superior to contrast-enhanced CT/MRI for tiny gastrointestinal NENs (61.5% *vs.* 37.5%). In the present study, the smallest diameter of gastrointestinal NEN detected by ^18^F-AlF-OC PET/CT was 0.6 cm in the rectum, 0.3 cm in the stomach, and 0.5 cm in the duodenum. Similarly, Chen *et al.* reported that the smallest diameter of NEN detected by ^18^F-AlF-OC PET/CT was 0.4cm in the rectum. These findings suggested that ^18^F-AlF-OC PET/CT is a useful imaging modality for the early diagnosis of tiny gastrointestinal NENs.

Impact on clinical management is a prerequisite for acceptance of any new diagnostic imaging modality by physicians. A meta-analysis evaluating the impact of ^68^Ga-SSTR PET/CT on the management of NEN patients included 14 studies and revealed that an overall change in management occurred in 44% of patients (range, 16% to 71%) 29. Analysis of our data revealed that ^18^F-AlF-OC PET/CT results led to a change in the therapeutic management in 19.5% of patients, mainly due to additional or unexpected findings. This result was within the range of 16% to 71% while lower than the overall change of 44% reported for ^68^Ga-SSTR PET/CT. We noticed that in several studies, a frequently reported impact of ^68^Ga-SSTR PET/CT on management was the initiation or continuance of PRRT 15, 16, 30-32. Srirajaskanthan *et al.* reported the highest management impact of ^68^Ga-SSTR PET/CT of 71% for NEN patients 15. They found that ^68^Ga-DOTATATE imaging changed the management in 36 of 51 patients. Of these 36, 20 were considered suitable for PRRT. In another study analyzing one of the largest cohorts of NEN patients examined with ^68^Ga-DOTATATE (n = 728), the treatment plan was changed in 40.9% of patients, and in most cases, the new treatment comprised chemotherapy or PRRT 32. As a matter of fact, many patients in our cohort were considered suitable candidates for PRRT according to their ^18^F-AlF-OC PET/CT findings. However, PRRT was recommended only in 2 patients. This was mainly due to the fact that PRRT is currently not available in routine clinical practice in our country. We have reason to believe that ^18^F-AlF-OC PET/CT is expected to have an even higher impact on the management of NEN patients when PRRT becomes more widely clinically available.

Currently, ^18^F-labeled SSTR ligands other than ^18^F-AlF-OC are emerging in the molecular imaging of NENs and should be further explored. Most notably, [^18^F]F-SiFAlin-TATE ([^18^F]SiTATE) represents a promising alternative for NEN imaging. Recently published first-in-human data reported favorable characteristics of [^18^F]SiTATE, including high image quality and high tracer uptake in most tumor lesions 33, 34. To further intricate the picture, ^18^F-labeled SSTR antagonists are currently under pre-clinical investigation 35. Our data on the use of ^18^F-AlF-OC PET/CT is encouraging. Further comparison of ^18^F-AlF-OC and other ^18^F-labeled SSTR ligands is warranted.

One of the important limitations of this study is the lack of histologic confirmation for all detected lesions, such confirmation was not possible for both ethical and practical reasons. Therefore, a lesion-based analysis was not performed, as verification for all lesions was difficult either by histology or radiological follow-up. In addition, the follow-up period was not long enough (range, 4.5∼14 months), which might potentially hamper the diagnosis in some cases. It should be noted that all tiny gastrointestinal NENs were confirmed histologically, providing strong evidence about the value of SSTR PET/CT in such cases for the first time. Secondly, the study population is very heterogeneous and includes patients with suspected NENs to patients with metastatic disease and all gradings of NENs. It is therefore difficult to interpret the reported sensitivity and specificity that is averaged over these very different disease states. Furthermore, ^68^Ga-DOTATATE is approved for diagnosis on NET and considered the gold standard for imaging NET. The fact that we did not compare ^18^F-AlF-OC with ^68^Ga-DOTATATE represents another major limitation of our study.

## Conclusion

^18^F-AlF-OC PET/CT exhibited great diagnostic performance in NEN patients and is useful in detecting tiny gastrointestinal NENs. Furthermore, ^18^F-AlF-OC PET/CT impacted the therapeutic management in 19.5% of patients. Overall, our results further validate the critical role of ^18^F-AlF-OC for somatostatin receptor imaging in clinical practice.

## Figures and Tables

**Table 1:** Demographic and clinical characteristics of patients (*n* = 205)

Characteristic	No. (%)
**Number of patients**	205
**Male**	111 (54.1%)
**Female**	94 (45.9%)
**Age (years)**	55 (20-94)
**Primary tumor site**	
Pancreas	46 (22.4%)
Stomach	39 (19.0%)
Rectum	31 (15.1%)
Small intestine	25 (12.2%)
Thymus	17 (8.3%)
Lung	15 (7.3%)
Cancer of unknown primary	9 (4.4%)
Paraganglioma	9 (4.4%)
Others	10 (4.9%)
**Tumor grade**	
G1	74 (36.1%)
G2	90 (43.9%)
G3	21 (10.2%)
NEC	6 (2.9%)
NA	1 (0.5%)
**Treatment**	
Surgery	98 (47.8%)
Somatostatin analogue	20 (9.8%)
Chemotherapy	38 (18.5%)
Targeted therapy	23 (11.2%)
Radiotherapy	10 (4.9%)
Transarterial chemoembolization	12 (5.9%)
Immunotherapy	3 (1.5%)

NA, not available

**Table 2:** Diagnostic performance of ^18^F-AlF-OC PET/CT and contrast-enhanced CT/MRI

Diagnostic performance	^18^F-AlF-OC PET/CT	Contrast-enhanced CT/MRI
**All patients**		
Sensitivity	90.5% (133/147)	81.8% (112/137)
Specificity	93.1% (54/58)	71.1% (27/38)
Diagnostic accuracy	91.2% (187/205)	79.4% (139/175)
**Tiny gastrointestinal NEN**		
Sensitivity (per-patient level)	61.5% (16/26)	37.5% (9/24)
Sensitivity (per-lesion level)	53.7% (22/41)	35.1% (13/37)

^18^F-AlF-OC, ^18^F-AlF-NOTA-octreotide; PET/CT, positron emission tomography/computed tomography; MRI, magnetic resonance imaging; NENs, neuroendocrine neoplasms

**Table 3:** Diagnostic value of ^18^F-AlF-OC PET/CT for different tumor grades

Grades	Diagnostic value of ^18^F-AlF-OC		
	Sensitivity	Specificity	Diagnostic accuracy
**Grade 1**	71.1% (32/45)	100% (29/29)	82.4% (61/74)
**Grade 2**	100% (68/68)	90.9% (20/22)	97.8% (88/90)
**Grade 3/NEC**	96% (24/25)	100% (2/2)	96.3% (26/27)

^18^F-AlF-OC,^ 18^F-AlF-NOTA-octreotide; NEC, neuroendocrine carcinoma

## Abbreviations

NENs: Neuroendocrine neoplasms
SSTRs: somatostatin receptors
SSAs: somatostatin analogs
SUVmax: maximal standardized uptake value
PACS: Picture Archiving and Communication Systems
[^18^F]SiTATE: [^18^F]F-SiFAlin-TATE
